# TMED3/RPS15A Axis promotes the development and progression of osteosarcoma

**DOI:** 10.1186/s12935-021-02340-w

**Published:** 2021-11-27

**Authors:** Wei Xu, Yifan Li, Xiaojian Ye, Yunhan Ji, Yu Chen, Xiangyang Zhang, Zhikun Li

**Affiliations:** Department of orthopedic, Tongren Hospital, Shanghai Jiao Tong University School of Medicine, 1111 Xianxia Road, Changning District, 200336 Shanghai, People’s Republic of China

**Keywords:** Osteosarcoma, TMED3, RPS15A, Proliferation, Apoptosis, Migration

## Abstract

**Background:**

Osteosarcoma is a primary malignant tumor that mainly affects children and young adults. Transmembrane emp24 trafficking protein 3 (TMED3) may be involved in the regulation of malignant cancer behaviors. However, the role of TMED3 in osteosarcoma remains mysterious. In this study, the potential biological function and underlying mechanism of TMED3 in progression of osteosarcoma was elaborated.

**Methods:**

The expression of TMED3 in osteosarcoma was analyzed by immunohistochemical staining. The biological function of TMED3 in osteosarcoma was determined through loss-of-function assays in vitro. The effect of TMED3 downregulation on osteosarcoma was further explored by xenograft tumor model. The molecular mechanism of the regulation of TMED3 on osteosarcoma was determined by gene expression profile analysis.

**Results:**

The expression of TMED3 in osteosarcoma tissues was significantly greater than that in matched adjacent normal tissues. Knockdown of TMED3 inhibited the progression of osteosarcoma by suppressing proliferation, impeding migration and enhancing apoptosis in vitro. We further validated that knockdown of TMED3 inhibited osteosarcoma generation in vivo. Additionally, ribosomal protein S15A (RPS15A) was determined as a potential downstream target for TMED3 involved in the progression of osteosarcoma. Further investigations elucidated that the simultaneous knockdown of RPS15A and TMED3 intensified the inhibitory effects on osteosarcoma cells. Importantly, knockdown of RPS15A alleviated the promotion effects of TMED3 overexpression in osteosarcoma cells.

**Conclusions:**

In summary, these findings emphasized the importance of TMED3/RPS15A axis in promoting tumor progression, which may be a promising candidate for molecular therapy of osteosarcoma.

**Supplementary Information:**

The online version contains supplementary material available at 10.1186/s12935-021-02340-w.

## Background

Osteosarcoma, also known as osteogenic sarcoma, is a highly aggressive primary tumor that is more common in children and young adults [1–3]. At present, the conventional treatment for osteosarcoma is usually surgical resection of primary or possibly metastatic tumors, plus systemic chemotherapy before and after surgery with drugs, such as doxorubin, cisplatin and methotrexate [4–6]. Unfortunately, this established treatment regimen has a high likelihood of short-term recurrence and significant long-term toxicity [7]. Whether primary or recurrent metastatic osteosarcoma patients, the cure rate is no more than 60–65% and the 5-year survival rate is less than 30% [3, 8, 9].

In view of the fact that no new treatment has been found to improve the cure rate of osteosarcoma patients, novel therapeutic markers, targeted drugs and innovative treatment strategies need to be identified [10]. Therefore, characterization and better understanding the new biomolecules or signal pathways involved in the procession of osteosarcoma was still of great significance.

Transmembrane emp24 trafficking protein 3 (TMED3), a member of the p24 protein family, is involved in the selection and secretion of COP vesicles in the endoplasmic reticulum-Golgi network and can affect multiple signaling pathways [11, 12]. Previous studies have indicated that TMED3 may be involved in the regulation of malignant cancer behaviors. TMED3 was initially found to be a metastatic inhibitory factor for colon cancer [13]. Moreover, Mishra et al., demonstrated that the secretory-transcription loop regulated by TMED3 and TMED9 is a key regulatory network to inhibit colon cancer metastatic [14]. In contrast, Zheng et al., reported that TMED3 can promote IL11 signal transduction and tumor progression in hepatocellular carcinoma cells [15]. Furthermore, Pei et al. illuminated that TMED3 promotes the proliferation and movement of breast cancer cells, which is negatively regulated by miR-188-3p [16]. In addition, TMED3 is clinically significant as a potential prognostic factor for renal cell carcinoma [17]. Interestingly, the role of TMED3 in malignancies appears to be different and even controversial. Although the diverse characters of TMED3 in tumor progression have been increasingly recognized in recent years, whose potential mechanism in osteosarcoma is still mystical.

In this study, we determined the overexpression of TMED3 in osteosarcoma tissues relative to normal tissues by IHC analysis and explored the relationship between TMED3 and clinicopathologic feature in patients with osteosarcoma. Moreover, a series of loss-of-function experiments revealed the promoting role of TMED3 on osteosarcoma cell growth, migration and tumor formation. Subsequently, the mechanism of TMED3 regulating osteosarcoma was analyzed by microarray analysis. Collectively, our study indicated that TMED3/RPS15A axis played vital roles in osteosarcoma progression and which might become a potential therapeutic target for osteosarcoma treatment.

## Methods

### Sample collection from patients with osteosarcoma

In this study, we purchased a tissue microarray chip consisting of osteosarcoma tissue (n = 70) and normal bone tissue (n = 1). Inclusion criteria: patients pathologically diagnosed with osteosarcoma by two experienced pathologists; tissues of patients with osteosarcoma who have only undergone surgical treatment; patients with osteosarcoma with corresponding medical records before and after surgery. Exclusion criteria: Patients with osteosarcoma treated with radiotherapy or chemotherapy. The study was approved by the Institutional Review Board of Tongren Hospital, School of Medicine, Shanghai Jiao Tong University and all patients had signed informed consent forms.

### Immunohistochemical (IHC) staining

The expression of TMED3 or RPS15A in osteosarcoma and adjacent normal bone tissues was observed by IHC staining, respectively. Firstly, the tissue sections were dewaxed, repaired with citric acid antigen and blocked by animal serum. Then, the tissue sections were first incubated with primary antibodies, and then with HRP-conjugated goat anti-rabbit IgG at 4 °C (The antibody information was shown in Additional file 6: Table S1). In the absence of light, the tissue sections were stained with DAB and hematoxylin (Baso, Cat. No. BA4022). Finally, the IHC scores were determined by staining percentage scores [classified as: 1 (1–24%), 2 (25–49%), 3 (50–74%), 4 (75–100%)] and staining intensity scores (scored as 0: signal less color, 1: brown, 2: light yellow, 3: dark brown). The IHC in each tissue section was assessed by at least two pathologists without knowledge of the clinical or pathological details of tumors, and a consensus score was finally reached for each core. According to the scores of IHC staining, we defined TMED3 as high expression if it is greater than the median, otherwise as low expression. The same is true for the evaluation criteria for high or low expression of RPS15A.

### Cell culture

Human osteoblast cells (hFOB 1.19) and osteosarcoma cell lines (U-2OS, Saos-2, MNNG/HOS, MG-63) were obtained from Cell Bank of the Chinese Academy of Sciences (Shanghai, China). All cell lines were maintained in Dulbecco’s modified Eagle’s medium (DMEM) supplemented with 10% FBS and 100 U/ml penicillin G and streptomycin and 1% nonessential amino acids, which were cultivated in a 37 °C incubator under the conditions of 5% CO2 and 95% humid air. In brief, U-2OS was taken from a cell line of a 15-year-old girl with a differentiated sarcoma of the tibia. MNNH/HOS comes from a 13-year-old girl suffering from osteosarcoma, histological morphology is mixed fibroblasts and epithelioid cells.

### Cell transfection, lentivirus production and infection

Firstly, the RNA interference (RNAi) target sequences were designed according to the gene sequences of TMED3 and RPS15A as templates (The target sequences were shown in Additional file 7: Table S2). Subsequently, the synthesized RNAi target sequence and scramble sequence (negative control) were ligated with BR-V-108 or LV-004 lentivirus vector (Shanghai Yiberui Biomedical Technology, Shanghai, China), respectively. The plasmids of recombinant vector BR-V-108/LV-004 and pack auxiliary vector (Helper 1.0 and Helper 2.0) were obtained and co-transfected into 293T cells. After 72 h of culture, the supernatant was collected and tested for lentivirus quality, such as viscosity, asepsis and titer. The 20 µl lentiviruses (1 × 10^8^ TU/ml) with target sequences and green fluorescent protein (GFP) tags were produced and used to infect MNNG/HOS and U-2OS (2 × 10^5^ cells/well) at 10 MOI, respectively. After 72 h of continuous culture, the GFP was observed under fluorescence microscope. Then, the stable cell lines were selected with puromycin as previously described [18].

Notably, in the rescue experiments, NC(OE+KD) group was the cells infected with empty vector (LV-004 and BR-V108), as negative control. TMED3 + NC(KD) group was TMED3 overexpressed cells. shRPS15A + NC(OE) group was RPS15A knockdown cells. TMED3 + shRPS15A group was cells that overexpressed TMED3 and knocked down RPS15A at the same time simultaneously. shRPS15A + shTMED3 was the cells that knockdown of RPS15A and TMED3 simultaneously.

### Quantitative PCR (qPCR)

Briefly, the total RNA of MNNG/HOS and U-2OS was extracted using Trizol reagent (Sigma, Cat. No. T9424-100 m). Subsequently, Hiscript QRT supermix (Vazyme, Nanjing, China, Cat. No. R123-01) was used for reverse transcription of RNA to obtain cDNA. The relative gene expression levels were calculated and statistically compared using the 2^–ΔΔCT^ analysis program and GAPDH was used as an internal control. The primer sequences were listed in Additional file 8: Table S3.

### Western blotting (WB)

The total proteins of MNNG/HOS and U-2OS were extracted with cell lysate, and detected by BCA protein detection kit (HyClone-Pierce). The 10-µg protein was separated by SDS-PAGE (Invitrogen) and transferred to the PVDF membrane, then sealed at room temperature for 1 h with TBST solution. After that, the membrane was first incubated with primary antibodies (Additional file 6: Table S1) overnight at 4 °C, and with HRP-conjugated goat anti-rabbit IgG for another 2 h at room temperature (The antibody information was shown in Additional file 6: Table S1). Finally, Millipore Immobilon Western Chemiluminescent HRP Substrote kit (Millipore, Cat. No. RPN2232) was used for color rendering and Chemiluminescent imager (GE, Cat. No. AI600) observation.

### MTT assay

The MNNG/HOS and U-2OS cells were laid in a 96-well plate with a density of 2000 cells per well. From the second day, the OD value (490/570 nm) was detected by Microplate Reader (Tecan infinite) at the same time every day for 5 days. Before the absorbance was detected, 5 mg/ml MTT (Genview, China, Cat. No. JT343) 20 µl was added to the hole for 4 h, and then 100 µl DMSO solution was added.

### Colony formation assay

The MNNG/HOS and U-2OS cells were cultured in a 6-well plate with a density of 800 cells per well. The cells were cultured in an incubator until the number of cells in most single clones was greater than 50. After 14 days, the cells were fixed with 4% paraformaldehyde 1 ml for 60 min. After that, the cells were stained with GIEMSA staining solution for 10-20 min, and then cell clones were photographed.

### Celigo cell counting assay

The MNNG/HOS cells were cultured in an incubator with a density of 2000 cells per well. From the second day, cells were counted by Celigo (Nexcelom) at the same time every day for consecutive 5 days. After that, the number of green fluorescent cells in each scanning orifice plate was accurately calculated, and the cell proliferation curve was drawn.

### Cell apoptosis analysis by flow cytometry

The MNNG/HOS and U-2OS cells were inoculated in 6-well plate and cultured continuously for 7 days. The cells were centrifuged at 1500 rpm for 5 min and washed with PBS and 1 × binding buffer (eBioscience, USA, Cat. No. 88-8007-74) respectively, and then the cell precipitation was resuspended in 1 ml of 1 × binding buffer. 100 µl of cell suspension (1 × 10^6^ cells) was stained with 5 µl of annexin V-APC (eBioscience) in the dark for 15 min, then centrifuged and resuspended in 1 × binding buffer. Subsequently, the cells were stained with 5 µl of PI (eBioscience), supplemented with buffer, and the apoptosis rate was tested by flow cytometry (Millipore).

### Wound-healing assay

The MNNG/HOS and U-2OS cells were cultured in 96-well plate at the density of 50,000 cells per well. After the low concentration of serum medium was replaced the next day, the scratches were formed by nudging upward at the center of the lower end of the 96-well plate with a scratch meter. Cellomics (Thermo) was performed to scan the 96-well plate when they were continuously cultured for 4 and 8 h. Finally, the migration area was calculated and analyzed under fluorescence-based Cellomics ArrayScan VTI analyzer (Thermo Fisher Scientific).

### Transwell assay

The cells MNNG/HOS and U-2OS were inoculated in a well-hydrated chamber (3422 corning) with a density of 50,000 cells per well. The inner chamber contained 100 µl of serum-free medium and the external chamber contained 600 µl 30% fetal bovine serum. After the cell suspension was diluted with serum-free medium and then cells were added to each chamber for 24 h cultivation. The migrating cells were fixed with 4% formaldehyde and photographed after Giemsa staining to analyze the cell migration rate. Finally, the cells were placed under a 200 × microscope (Olympus) to capture images from five randomly selected fields.

### Mouse xenograft model

The 4-week-old female BALB/c nude mice (Shanghai Lingchang Biotechnology) were randomly divided into different groups (shCtrl vs. shTMED3, n = 10 per group) for tumor xenograft assays. The procedures were displayed approval by the Institutional Review Board of Tongren Hospital, School of Medicine, Shanghai Jiao Tong University. MNNG/HOS cells (4 × 10^6^ cells/ml) were injected subcutaneously into the right forelimb axillary in nude mice. The mice were anesthetized by intraperitoneal injection of 0.7% pentobarbital sodium (Sigma, Cat. No. P3761) at the dose of 40 mg/kg and then imaged under a living imager and the total fluorescence intensity was recorded. Tumor size and animal weight were measured every other day 10 days after subcutaneous injection. Tumor volumes (in mm^3^) were measured use a caliper and calculated by a formula: π/6 × L × W^2^, where L is the long axis and W is the short axis of tumor. 23 days later, the mice were euthanized at the dose of 100 mg/kg using pentobarbital and the tumors were removed, weighed and photographed for retention. The mice tumors were fixed with 10% formalin, soaked in xylene and ethanol in turn, and then blocked with 3% PBS-H_2_O_2_. After that, the tissue sections were first incubated with antibody Ki67, and then incubated with HRP goat anti-rabbit IgG (The antibody information was shown in Additional file 6: Table S1). In the absence of light, the tissue sections were stained with hematoxylin (Baso, Cat. No. BA4022) and DAB. Notably, the same method was applicable to the in vivo recovery experiments in mice (NC(OE+KD), TMED3+NC(KD), shRPS15A+NC(OE), TMED3+shRPS15A, n = 6 per group).

### Human apoptosis antibody array

The expression of apoptosis-related proteins was detected in MNNG/HOS cells using Human Apoptosis Antibody Array-Membrane kit (Abcam, USA, Cat. No. ab134001) according to the manufacturer’s protocols. After the MNNG/HOS cell protein was blocked by the special membrane of the antibody array, the buffer was used to wash the membrane to reduce the interference of the background signal intensity. Following, the membrane was incubated with the detection antibody Cocktail (1:100) for an hour and then linked to streptavidin conjugate with HRP. Finally, the spots were visualized with enhanced chemiluminescence (ECL) (Amersham) and then the gray value were analyzed with ImageJ software (National Institute of Health). The apoptosis-related protein primer kit we used in this experiment has two spots for each protein. The statistical analysis of the results was based on the gray values of these two points being analyzed 3 times.

### Genechip prime view

Affymetrix human Gene Chip Prime View combined with Affymetrix Scanner 3000 scan was performed to analyze the effects of TMED3 knockdown on downstream gene expression levels. Accordingly, the scatter plot, volcano plot and hierarchical clustering of the shTMED3 and shCtrl in MNNG/HOS were presented by the differentially expressed genes (DEGs) with criterion of |Fold Change| ≥ 2.0 and false discovery rate (FDR) ≤ 0.05. Furthermore, the significant enrichment of DEGs in classical pathways as well as disease and function were demonstrated based on Ingenuity Pathway Analysis (IPA) analysis.

### Statistical analysis

All statistical analyses were performed with SPSS version 23.0 software (Chicago, IL, USA). Each experiment in this study was independently repeated at least three times to obtain the average values. Data were presented as the mean ± standard deviation. Statistical differences between two groups were evaluated using the unpaired Student’s t-test. The Chi-square test or Fisher exact probability analysis was used to analyze the associations between TMED3 or RPS15A expression and clinicopathological features of osteosarcoma. The correlation between TMED3 or RPS15A expression was evaluated by Mann-Whitney U test and Spearman correlation analysis. Survival curves were obtained by the Kaplan–Meier method, and differences in survival rates were assessed by the log-rank test. All statistical tests were two-tailed and values of P < 0.05 were considered statistically significant.

## Results

### TMED3 is highly expressed in osteosarcoma tissue

**Fig. 1:**
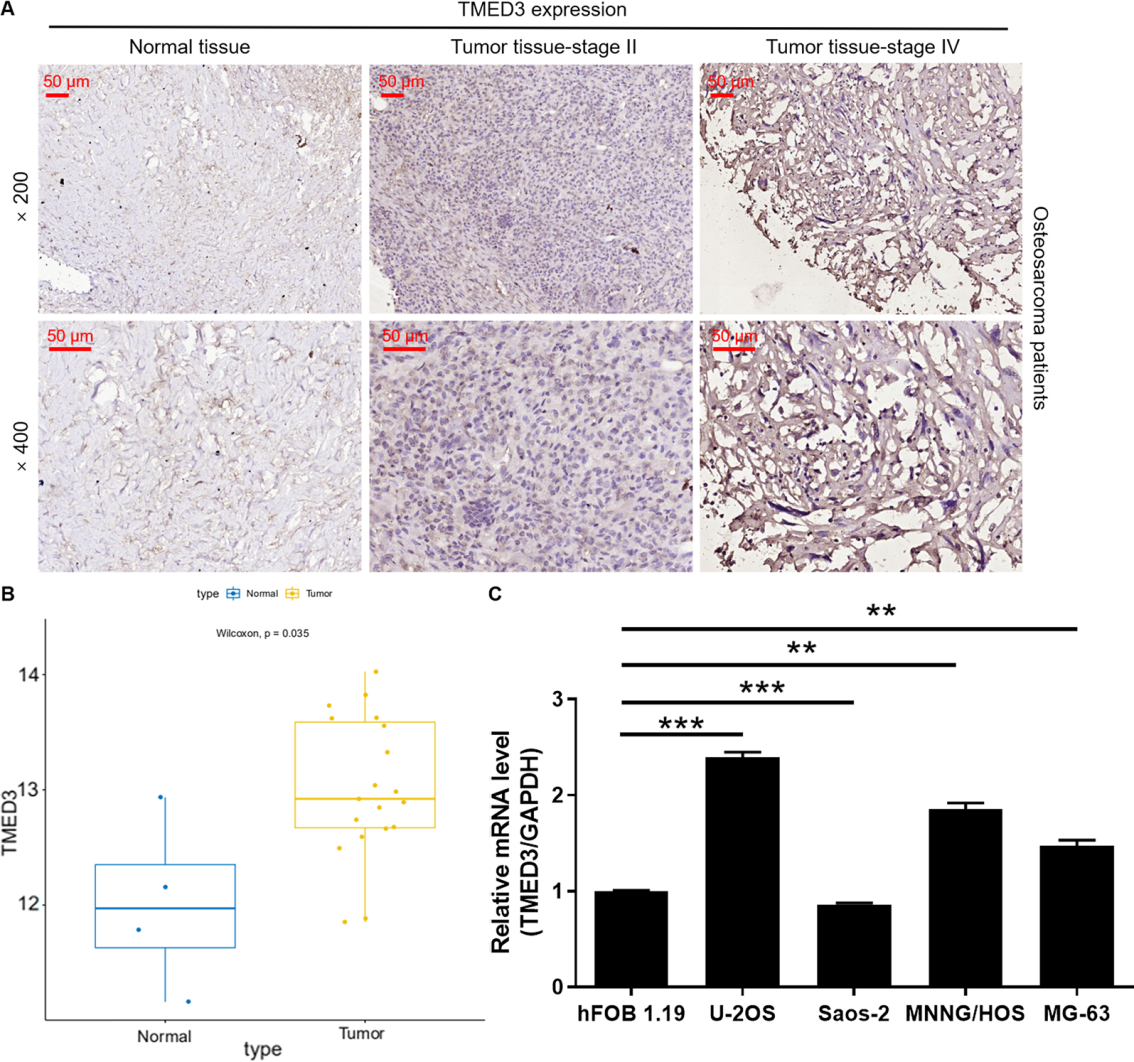
TMED3 is overexpressed in osteosarcoma. **A** The representative picture of the expression level of TMED3 in normal tissues and tumor tissues with different grade was detected by immunohistochemistry (IHC). The magnification is 200. **B** TMED3 expression profiles of tumor (n = 19) and normal (n = 4) samples from osteosarcoma patients was analyzed from GEO database. **C** The mRNA expression of TMED3 in human osteosarcoma cells (U-2OS, Saos-2, MNNG/HOS, MG-63) and osteoblast cells (hFOB 1.19) was accessed by qPCR. The presented figures are representative data from at least three independent experiments

According to the scores of IHC, we defined TMED3 as high expression if it is greater than the median, otherwise as low expression. The expression of TMED3 in osteosarcoma was significantly higher than that in normal tissues (P < 0.001) (Table 1). The representative pictures of IHC staining can more intuitively find that the number of stained cells in osteosarcoma was remarkably more than that in normal tissues (Fig. 1A). Based on the GEO database, we further analyzed that TMED3 expression profiles of tumor and normal samples from osteosarcoma patients. As showed in Fig. 1B, TMED3 was highly expressed in osteosarcoma tissue. Moreover, osteosarcoma patients with high expression level of TMED3 had shorter survival period. However, perhaps due to the sample size of the TARGET-OS database, there is no significant correlation between them (Additional file 1: Fig. S1A). Consistently, the expression levels of TMED3 in human osteosarcoma cells (U-2OS, Saos-2, MNNG/HOS, MG-63) was higher than the osteoblast cells hFOB 1.19 (Fig. 1C). The subsequent statistical analysis of the Mann-Whitney U (Table 2) and Spearman correlation (Table 3) showed that there was a significant positive correlation between the level of TMED3 and pathological data such as T Infiltrate, lymphatic metastasis and Stage. The increased expression of TMED3 indicated the deepening of tumor malignancy in patients with osteosarcoma. Taken together, TMED3 was not only highly expressed in patients with osteosarcoma, but also positively correlated with the malignancy degree.

**Table 1:** Expression patterns in gastric cancer tissues and normal tissues revealed in immunohistochemistry analysis

TMED3 expression	Tumor tissue		Normal tissue		P value
	Cases	Percentage	Cases	Percentage	
Low	34	61.8%	7	100.0%	0.003
High	21	38.2%	0	0%	

**Table 2:** Relationship between TMED3 expression and tumor characteristics in patients with osteosarcoma

Features	No. of patients	TMED3 expression		P value
		Low	High	
All patients	55	34	21	
Age (years)				
< 28	26	15	11	0.555
≥ 28	29	19	10	
Gender				
Male	37	23	14	0.941
Female	18	11	7	
Tumor size				
< 6	11	8	3	0.395
≥ 6	26	15	11	
T infiltrate				
T1	27	21	6	0.007
T2	25	13	12	
T3	3	0	3	
lymphatic metastasis (N)				
N0	52	34	18	0.025
N1	3	0	3	
Stage				
1	1	1	0	0.021
2	51	33	18	
4	3	0	3	
RPS15A				
0	27	15	12	0.182
1	23	17	6	

**Table 3:** Relationship between TMED3 expression and tumor characteristics in patients with osteosarcoma

	TMED3
T Infiltrate	
Spearman correlation	0.367
Significance (two tails)	0.006
N	55
Lymphatic metastasis (N)	
Spearman correlation	0.306
Significance (two tails)	0.023
N	55
Stage	
Spearman correlation	0.314
Significance (two tails)	0.019
N	55

### Knockdown of TMED3 inhibits the progression of osteosarcoma in vitro

**Fig. 2:**
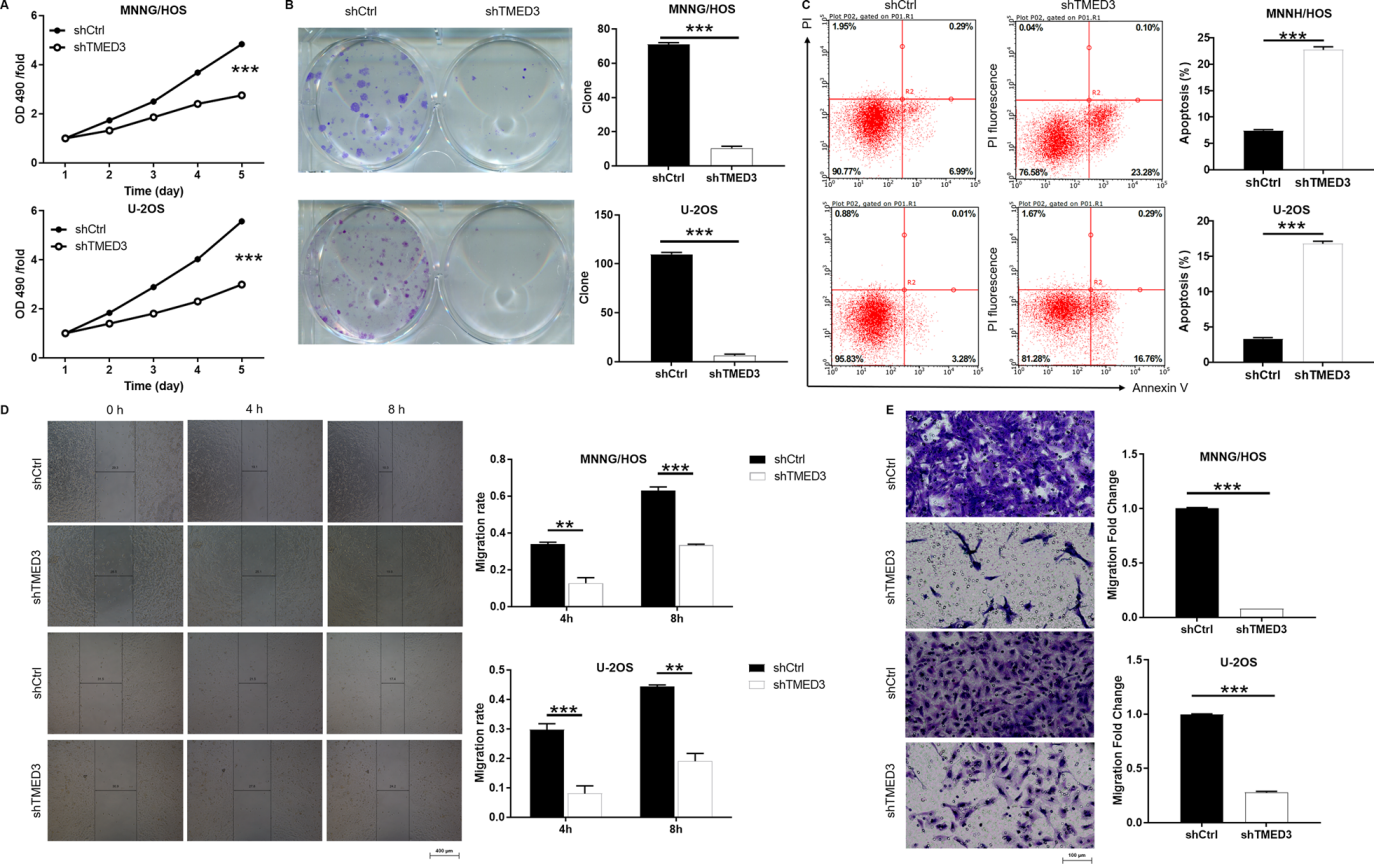
TMED3 knockdown inhibits progression of osteosarcoma in vitro. **A** MTT assay was employed to show the effects of TMED3 on cell proliferation of MNNG/HOS and U-2OS cells. **B**) Colony forming ability of MNNG/HOS and U-2OS cells was detected. **C** Flow cytometry was performed to detect cell apoptosis of MNNG/HOS and U-2OS cells with or without TMED3 knockdown. **D**, **E** The MNNG/HOS and U-2OS cell migration ability was accessed by wound-healing assay (**D**) and Transwell assay (**E**). The presented figures are representative data from at least three independent experiments. Data was shown as mean ± SD. *P < 0.05, **P < 0.01, ***P < 0.001

To elucidate the detailed function of TMED3/RPS15A in osteosarcoma, a cell model of TMED3 downregulation (shTMED3) was designed using lentivirus. Firstly, more than 80% of the green fluorescence signals observed under the microscope indicated successful infection of MNNG/HOS and U-2OS (Additional file 1: Fig. S1B). Secondly, the results of qPCR (Additional file 1: Fig. S1C) and WB (Additional file 1: Fig. S1D) presented the downregulation of TMED3 at mRNA and protein levels, respectively, suggesting that TMED3 was successfully knocked down in MNNG/HOS and U-2OS. The proliferation, clone number, apoptosis and migration ability of osteosarcoma cells were estimated to evaluate whether TMED3 is involved in the regulation of osteosarcoma. First of all, the MTT results showed that the value of OD490/fold in shTMED3 group was much lower than that in shCtrl group, which reflected that the knockdown of TMED3 weakened the cell viability (Fig. 2A). At the same time, the Fig. 2B indicated that the knockdown of TMED3 reduced the clone formation ability of MNNG/HOS and U-2OS cells. Moreover, flow cytometry detection of apoptosis showed that the downregulation of TMED3 resulted in a sharp increase in the apoptosis rate of MNNG/HOS and U-2OS (Fig. 2C). Moreover, the preliminary molecular mechanism by which TMED3 knockdown induced apoptosis in osteosarcoma cells was elucidated using human apoptotic antibody array. As shown in Figure S1E, the expression of Caspase3 was increased. On the contrary, Bcl-2, Bcl-w, clAP-2, HSP27, IGF-I, IGF-1sR, sTNF-R1, sTNF-R2, TNF-α, TNF-β, TRAILR-3, TRAILR-4 and XIAP was decreased. In addition, wound-healing combined with Transwell assay both showed that the knockdown of TMED3 significantly inhibited the migration ability of MNNG/HOS and U-2OS cells (Fig. 2D, E). Comprehensive analysis validated that downregulation of TMED3 inhibited the progression of osteosarcoma by inhibiting proliferation, migration and enhancing apoptosis *in vitro*.

### Knockdown of TMED3 attenuates osteosarcoma generation in vivo

**Fig. 3:**
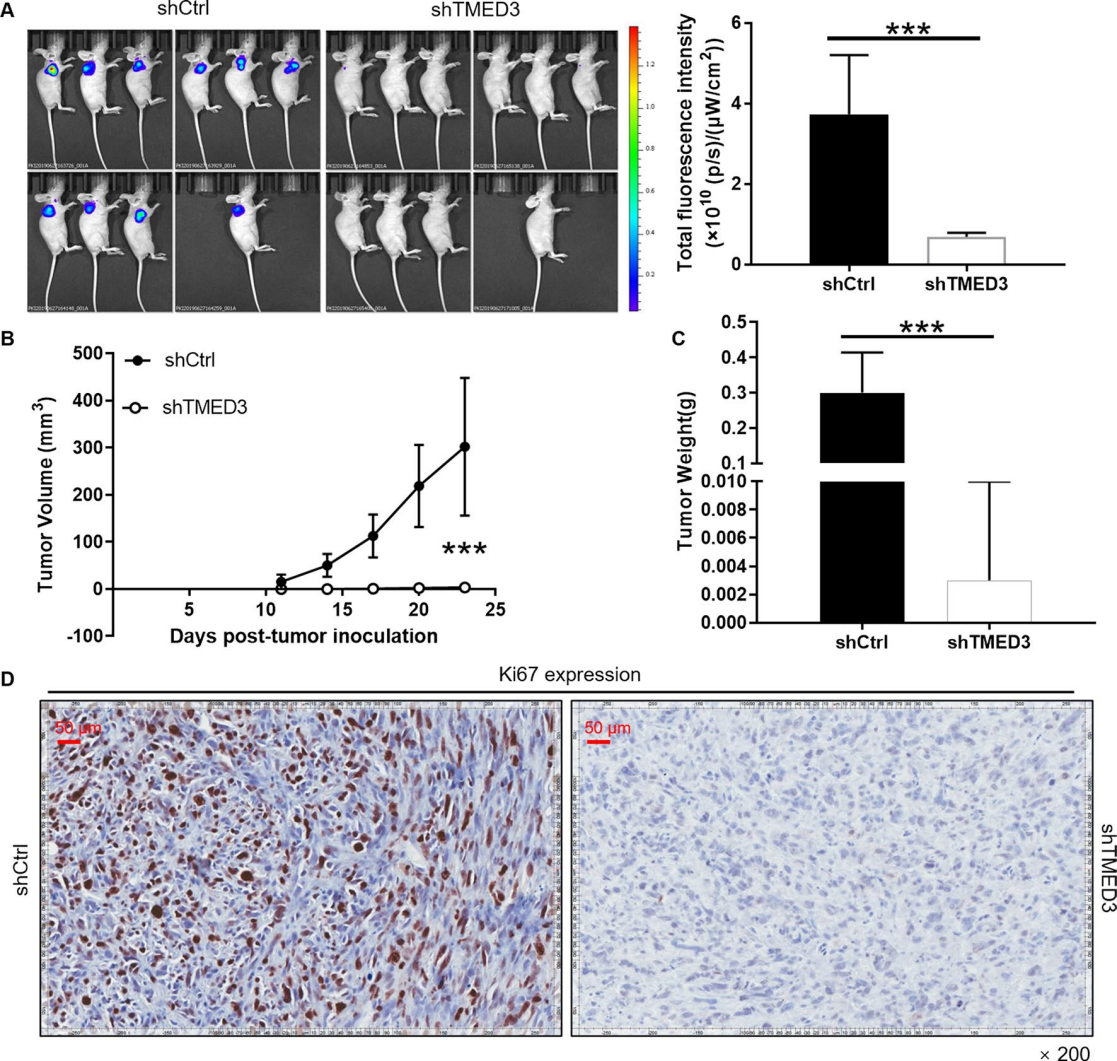
TMED3 knockdown suppress osteosarcoma development in vivo. **A** In vivo imaging was performed to evaluate the tumor burden in mice of shTMED3 and shCtrl groups post tumor-inoculation; The fluorescence intensity was scanned and used as a representation of tumor burden in mice of shTMED3 and shCtrl groups. **B**, **C** MNNG/HOS cells with or without TMED3 knockdown, the volume (**B**) and weight (**C**) of tumors formed in mice was measured and calculated at indicated time intervals. (D) The Ki67 level in tumors removed from mice was detected by IHC as a representation of tumor growth. Data was shown as mean ± SD. *P < 0.05, **P < 0.01, ***P < 0.001

The xenograft model was applied to further explore whether the downregulation of TMED3 can produce the same effects in vivo as in vitro. The fluorescence imaging results displayed that the tumor burden and the fluorescence intensity were reduced in shTMED3 group than those in shCtrl (Fig. 3A). After 23 days of monitoring, downregulation of TMED3 was found to cause the tumor in mice to be weaker than that of the control group in terms of volume (Fig. 3B) and weight (Fig. 3C). In addition, the tumor contrast photos more intuitively showed that the downregulation of TMED3 weakens the tumor growth in mice. Low positive cells with Ki67 staining were detected in shTMED3 tumor sections, further confirming the rationality of the above observations (Fig. 3D). Taken together, the downregulation of TMED3 could inhibited the osteosarcoma formation *in vivo*.

### RPS15A is a downstream target for TMED3 regulation of osteosarcoma

**Fig. 4:**
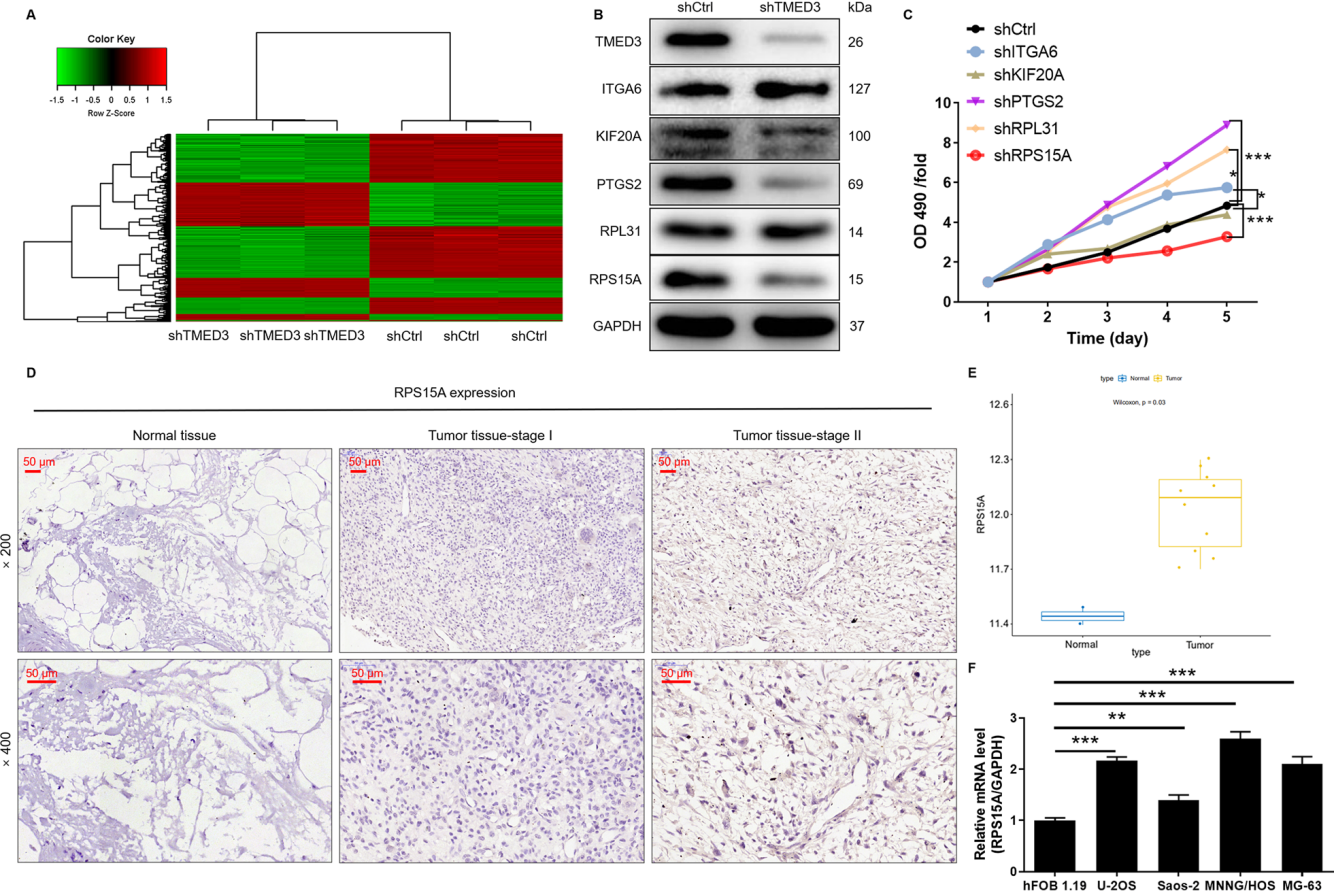
The exploration of downstream underlying TMED3 induced regulation of osteosarcoma. **A** A Prime View Human Gene Expression Array was performed to identify the differentially expressed genes (DEGs) between shTMED3 and shCtrl groups of MNNG/HOS cells. In the heat map of cluster analysis, each column represents a sample and each row represents a differential gene. The red indicates that the gene expression is upregulated, the green indicates that the gene expression is downregulated, the black indicates that the gene expression is not significantly changed, and the gray indicates that the signal strength of the gene is not detected. **B** The top 5 DEGs with the largest variation were screened out for further verification by WB. **C** The lentiviral system mediated knockdown of ITGA6, KIF20A, PTGS2, RPL31, RPS15A and tested the effects on the viability of osteosarcoma cells. **D** The expression of RPS15A in tumor tissues and normal tissues was evaluated by IHC analysis. **E** RPS15A expression profiles of tumor (n = 10) and normal (n = 2) samples from osteosarcoma patients was analyzed from GEO database. **F** The mRNA expression of RPS15A in human osteosarcoma cells (U-2OS, Saos-2, MNNG/HOS, MG-63) and osteoblast cells (hFOB 1.19) was accessed by qPCR. The representative images were selected from at least 3 independent experiments. Data was shown as mean ± SD. *P < 0.05, **P < 0.01, ***P < 0.001

The potential mechanism of TMED3 in osteosarcoma cells was further explored. The DEGs between group of shTMED3 and shCtrl in MNNG/HOS were identified through Genechip. The DEGs consisted of 291 upregulated and 487 downregulated, which were shown by scatter plot (Additional file 2: Fig. S2A), volcano plot (Additional file 2: Fig. S2B) and hierarchical clustering (Fig. 4A). In addition, the typical signal pathways (Figure S2C), diseases and functions (Additional file 2: Fig. S2D) of these DEGs enrichment functions in MNNG/HOS were analyzed according to IPA. Through gene chip analysis, the top 5 DEGs with the largest variation were screened out for further verification by WB. PTGS2 and RPS15A protein expressions were most significantly downregulated (Fig. 4B). Subsequently, the lentiviral system mediated knockdown of these 5 genes and tested the effect on the viability of osteosarcoma cells. Compared with other groups, knockdown of RPS15A inhibited the viability of osteosarcoma cells most significantly (Fig. 4C). Through IHC analysis, we found that the expression of RPS15A in tumor tissues of osteosarcoma was significantly higher than that of normal tissues adjacent to the cancer (Fig. 4D). Of note, the analysis results of the TARGET-OS database showed that RPS15A was abundantly expressed in osteosarcoma (Fig. 4E). Moreover, there is no significant correlation between RPS15A expression and the survival period in patients with osteosarcoma (Additional file 3: Fig. S3A). On the other hand, the mRNA expression levels of RPS15A in human osteosarcoma cells (U-2OS, Saos-2, MNNG/HOS, MG-63) was higher than the osteoblast cells hFOB 1.19 (Fig. 4F). Combined with the above data, we preliminarily considered that RPS15A was the downstream target of TMED3 regulation of osteosarcoma.

### Knockdown of RPS15A alleviates the promotion effects of TMED3 overexpression in osteosarcoma cells

**Fig. 5:**
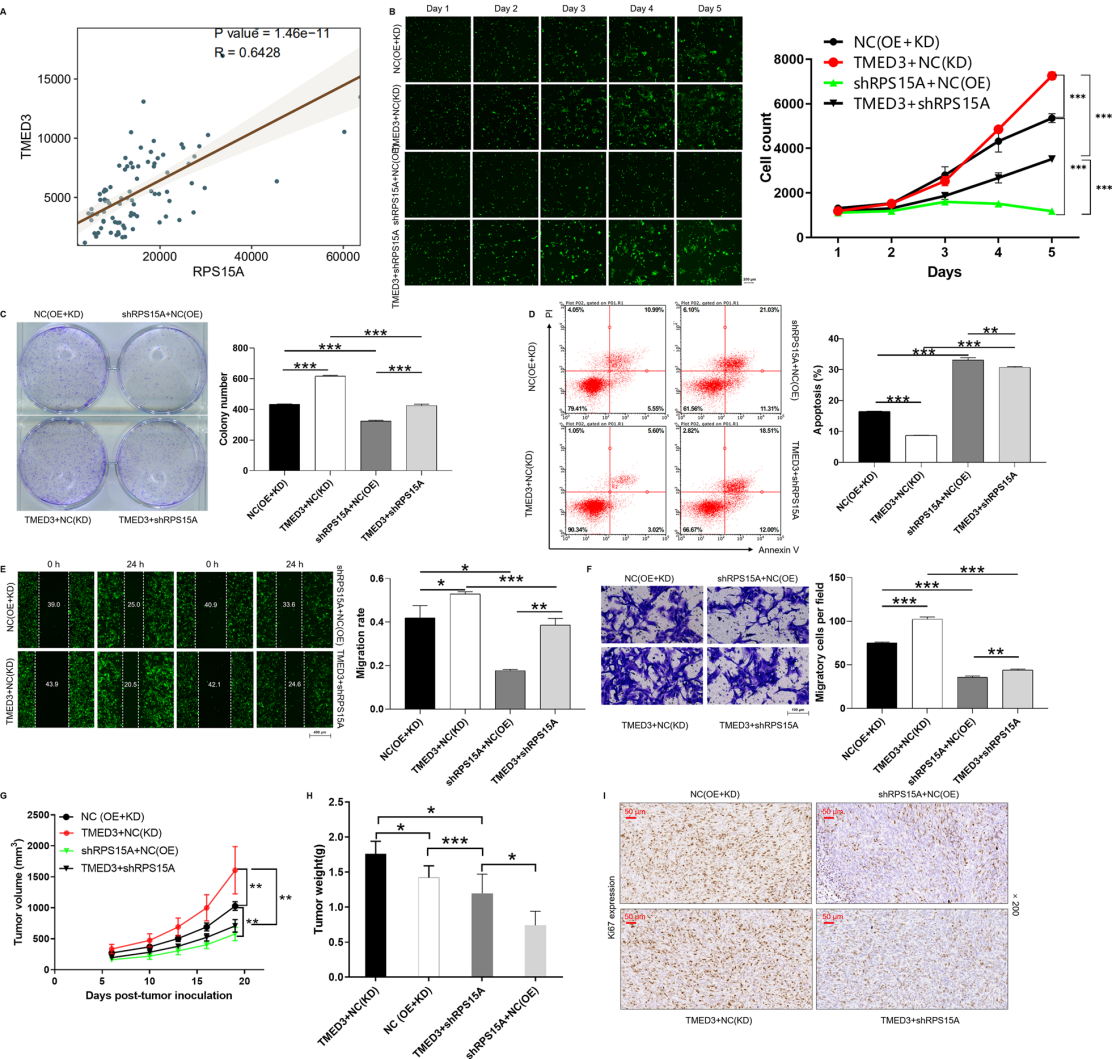
Overexpression of RPS15A alleviates the promotion effects of TMED3 knockdown in TNBC cells. **A** According to TARGET-OS database, the correlation between RPS15A and TMED3 expression in osteosarcoma was analyzed by Spearman Correlation Coefficient. Cell models were subjected to the detection of proliferation (**B**), colony (**C**) and apoptosis (**D**). The cell migration ability was accessed by wound-healing assay (**E**) and Transwell assay (**F**). BALB/c nude mice were randomly divided into NC (OE + KD), TMED3 + NC (KD), shRPS15A + NC (OE), TMED3 + shRPS15A; n = 6 per group) and detected the growth and expression of KI67 in mice. The representative images were selected from at least 3 independent experiments. Data was shown as mean ± SD. *P < 0.05, **P < 0.01, ***P < 0.001

Accordingly, the relationship between RPS15A and TMED3 in osteosarcoma aroused our interest. According to the TARGET-OS database, the expression of RPS15A and TMED3 in osteosarcoma was significantly positively correlated through Spearman analysis (Fig. 5A). Further investigation was required to determine whether RPS15A could regulate osteosarcoma progression. Analogously, in order to determine the effects of RPS15A and TMED3 on osteosarcoma, experimental groups of TMED3 + NC-shRPS15A, shRPS15A + NC-TMED3, TMED3 + shRPS15A and shRPS15A + shTMED3 were established in MNNG/HOS cells (Additional file 3: Fig. S3B–D). Subsequent the loss/gain of function experiments showed that knockdown of RPS15A alleviated the promotion effects of TMED3 overexpression in osteosarcoma cells, especially in proliferation (Fig. 5B), colony formation (Fig. 5C), apoptosis (Fig. 5D) and migration (Fig. 5E, F). Consistently, the rescue experiments in mice were performed and indicated the same effects (Fig. 5G–I). Moreover, MNNG/HOS cells models with deletion of RPS15A (ShRPS15A) and RPS15A + TMED3 (shRPS15A + shTMED3) were established, respectively (Additional file 4: Fig. S4A–D). The loss-of-function assays explained that the inhibitory effects of RPS15A knockdown on the progression of osteosarcoma cells was similar to that of TMED3. The simultaneous knockdown of RPS15A and TMED3 intensified the inhibitory effects on osteosarcoma cells in terms of proliferation, apoptosis and migration (Additional file 5: Fig. S5A–E). These results suggested that TMED3/RPS15A axis may play a role in promoting the development and progression of osteosarcoma.

## Discussion

Osteosarcoma still poses a significant health threat to adolescents [19]. Despite ongoing efforts to improve the diagnosis and prognosis of patients with osteosarcoma, current clinical therapies are far from meeting the need for full recovery, especially for patients with advanced phenotypes at the time of diagnosis [20, 21]. Therefore, there is an urgent need to identify promising genetic biomarkers as therapeutic targets and further discover new potential drugs for the treatment of osteosarcoma. Recently, the relationships between p24 family members and tumor progression have attracted much attention [14, 22, 23]. As a divergent member of the transmembrane trafficking protein family, TMED3 can promote signal transduction and regulate a variety of tumor cells progression [24–27]. Therefore, TMED3 is the subject of the design of potent anticancer drugs, and considered as a promising cancer therapeutic target [17, 24, 28]. In this study, our attention firstly focused on TMED3 expression in osteosarcoma. Combined with GEO database and immunohistochemical staining on the tissue microarray chip, identified that TMED3 is overexpressed in osteosarcoma. Moreover, there was a significant positive correlation between the expression level of TMED3 and pathological data such as T Infiltrate, lymphatic metastasis and stage. These findings indicated that TMED3 played crucial roles in osteosarcoma and could be used as a novel biomarker for the osteosarcoma diagnose.

Furthermore, the role of TMED3 in different types of tumors seems to be opposite, even controversial. For instance, TMED3 was initially found to be a metastatic inhibitory factor for colon cancer [13]. Recently, accumulating evidence reported that TMED3 exerts a protumor function in non-small cell lung cancer, lung squamous cell carcinoma and chordoma cells [25, 27, 29]. In the present study, we found that knockdown of TMED3 inhibited the behaviors of osteosarcoma cells on inhibiting proliferation, impeding migration and enhancing apoptosis. Collectively, our data showed that TMED3 acted as a tumor promoter in osteosarcoma.

Besides, the preliminary molecular mechanism by which TMED3 knockdown induced apoptosis in osteosarcoma cells was initially explored. It was well known that apoptosis is a complex and multi-step process, which required a synergistic effect of pro-apoptotic proteins, anti-apoptotic proteins and inhibitor of apoptosis proteins (IAPs) [30]. The present study found that knockdown of TMED3 could upregulated Caspase3 expression in osteosarcoma cells. On the contrary, Bcl-2, Bcl-w, clAP-2, sTNF-R1, sTNF-R2, TNF-α, TNF-β, TRAILR-3, TRAILR-4, IGF-I, IGF-1sR, HSP27 and XIAP was decreased. As we all known, Caspase-3 as a pro-apoptotic protein, is involved in the induction of apoptosis [31]. Bcl-2 family proteins, such as Bcl-2, Bcl-w and clAP-2 combine with tumor suppressors to promote apoptosis [32]. Furthermore, the occurrence of apoptosis needs the participation of anti-apoptosis proteins, which including tumor necrosis factors (TNF) (TNF-α, TNF-β), tumor necrosis factor-related apoptosis-inducing ligand (TRAIL), TRAIL receptor (TRAIL-R) (TRAILR-3, TRAILR-4), soluble TNF receptors type 1 (sTNF-R1), type 2 (sTNF-R2), insulin-like growth factor-I (IGF-I), relevant receptors IGF-1sR and heat shock protein (HSP27) [33–38]. Moreover, X-linked inhibitor of apoptosis (XIAP) plays an important role in preventing apoptotic cell death [39]. Collectively, TMED3 is involved in the initiation of events that regulate apoptosis, but the specific mechanism still needs to be further explored.

To profoundly investigate the function of TMED3 in osteosarcoma and preliminarily exploration its underlying mechanisms, a bioinformatics analysis was performed, followed by series of experiments to verify our findings from the gene microarray analysis. TMED3 was identified as a potential target of Ribosomal protein S15A (RPS15A) among the regulated downstream genes in osteosarcoma. Of note, a significantly positive correlation was recognized between the expressions of TMED3 and RPS15A. RPS15A is a highly conserved 40 S ribosomal protein located at the 16p12.3 site of human chromosome [40], which is indispensable in the assembly and translation of ribosomes [41]. In addition, RPS15A is responsible for maintaining a variety of biological functions, such as cell division, tumorigenesis and progression [42, 43]. A large amount of evidence demonstrated that RPS15A is abnormally highly expressed in various types of human cancers, including lung adenocarcinoma, hepatic cancer, colorectal cancer, breast cancer and osteosarcoma [44–48]. Consistently, the present study determined the abundant expression of RPS15A in osteosarcoma. Furthermore, RPS15A is involved in the regulation of a variety of cancers, including glioblastoma, gastric cancer, lung cancer, hepatocellular carcinoma and pancreatic cancer [49–55]. These findings suggested that the simultaneous knockdown of RPS15A and TMED3 intensified the inhibitory effects on osteosarcoma cells. More importantly, knockdown of RPS15A alleviated the promotion effects of TMED3 overexpression in osteosarcoma cells. Therefore, we suspected that TMED3 may modulate the apoptotic signaling via targeting RPS15A and regulating its activity in osteosarcoma, which certainly still warrants our further investigation.

## Conclusions

In conclusion, we have revealed a vital role for TMED3 in osteosarcoma, and our clinical analyses point the prospect of TMED3 as a diagnostic marker for osteosarcoma. Furthermore, knockdown of RPS15A alleviated the promotion effects of TMED3 overexpression in osteosarcoma cells. Therefore, TMED3/RPS15A axis may play a role in promoting the development and progression of osteosarcoma, which may provide a blueprint for the therapeutic target of osteosarcoma.

## Supplementary Information

**Additional file 1: Figure S1.** Effects of TMED3 expression level on survival and apoptosis-related proteins in osteosarcoma.

**Additional file 2: Figure S2.** Exploration of the effect of TMED3 knockdown on downstream signaling pathway and functional enrichment of osteosarcoma by Gene chip and Ingenuity Pathway Analysis (IPA).

**Additional file 3: Figure S3.** The effect of the expression level of RPS15A on the survival of osteosarcoma and the establishment of osteosarcoma cells that knock down RPS15A and TMED3 overexpression.

**Additional file 4: Figure S4.** Establishment of osteosarcoma cells knocked down RPS15A and TMED3.

**Additional file 5: Figure S5.** The effect of knockdown of RPS15A and TMED3 on the proliferation, apoptosis and migration of osteosarcoma cells.

**Additional file 6.** Antibodies used in IHC. Antibodies used in WB.

**Additional file 7.** The target sequences and shRNA sequences.

**Additional file 8.** Primersused in qPCR.

## Data Availability

The datasets used and/or analyzed during the current study are available from the corresponding author on reasonable request.
