# Supplementary figures and images for: TMED3/RPS15A Axis promotes the development and progression of osteosarcoma
Source: Cancer Cell Int. 2021 Nov 27;21:630. doi: 10.1186/s12935-021-02340-w (PMC8626936; doi:10.1186/s12935-021-02340-w)

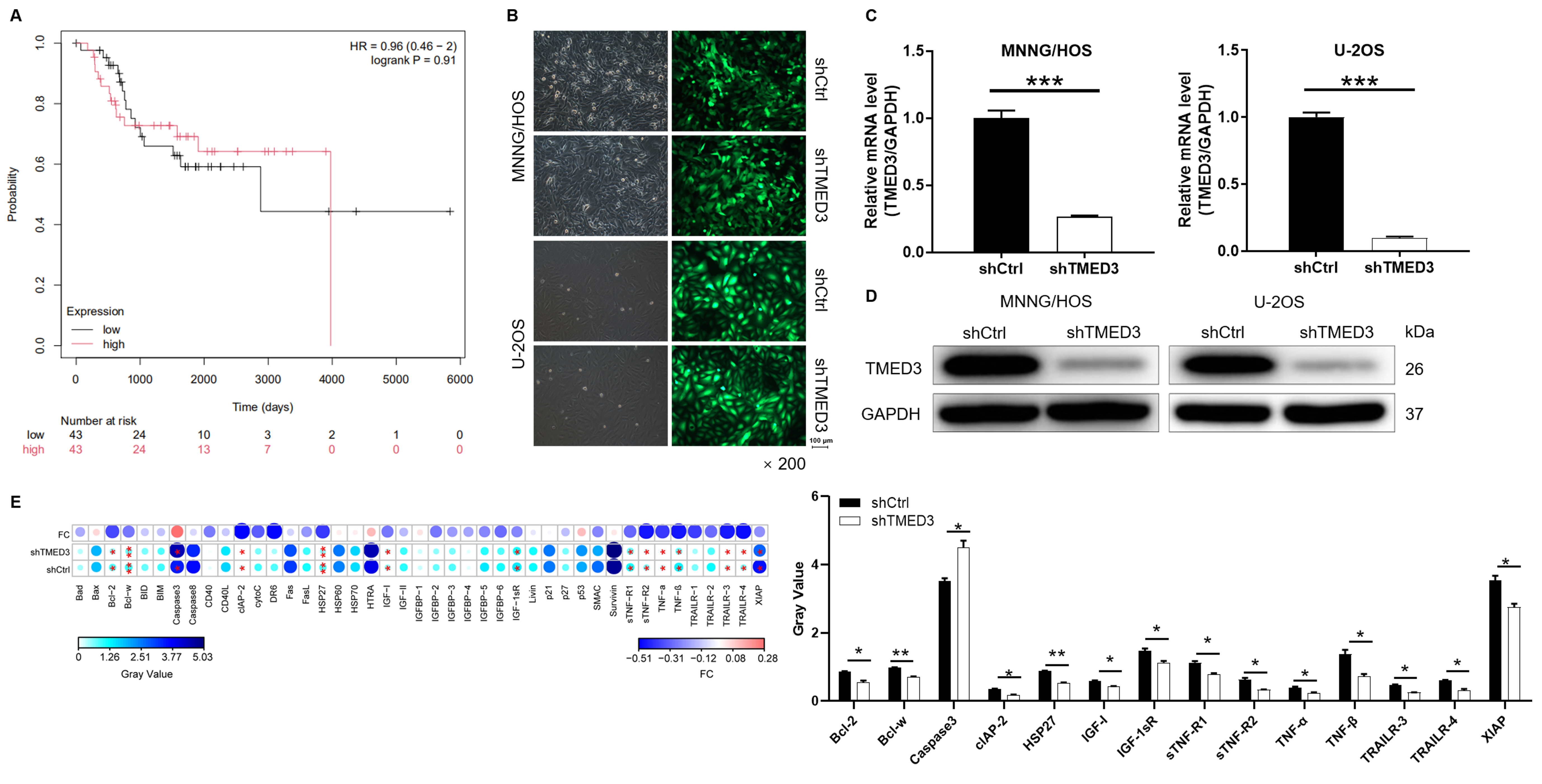

Supplement: Supplementary file 1 — Additional file 1: Figure S1. Effects of TMED3 expression level on survival and apoptosis-related proteins in osteosarcoma. [file 12935_2021_2340_MOESM1_ESM.tif]

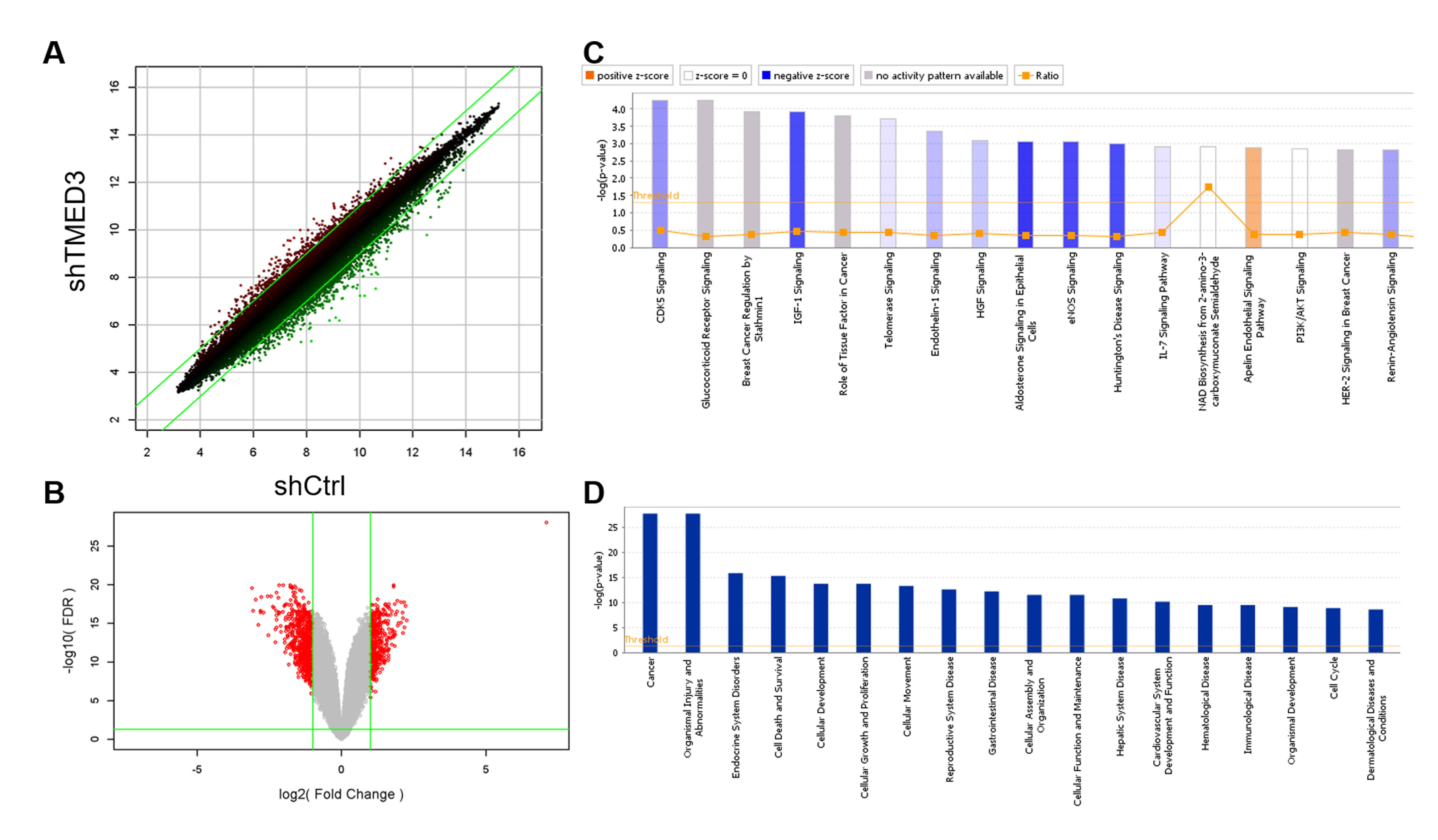

Supplement: Supplementary file 2 — Additional file 2: Figure S2. Exploration of the effect of TMED3 knockdown on downstream signaling pathway and functional enrichment of osteosarcoma by Gene chip and Ingenuity Pathway Analysis (IPA). [file 12935_2021_2340_MOESM2_ESM.tif]

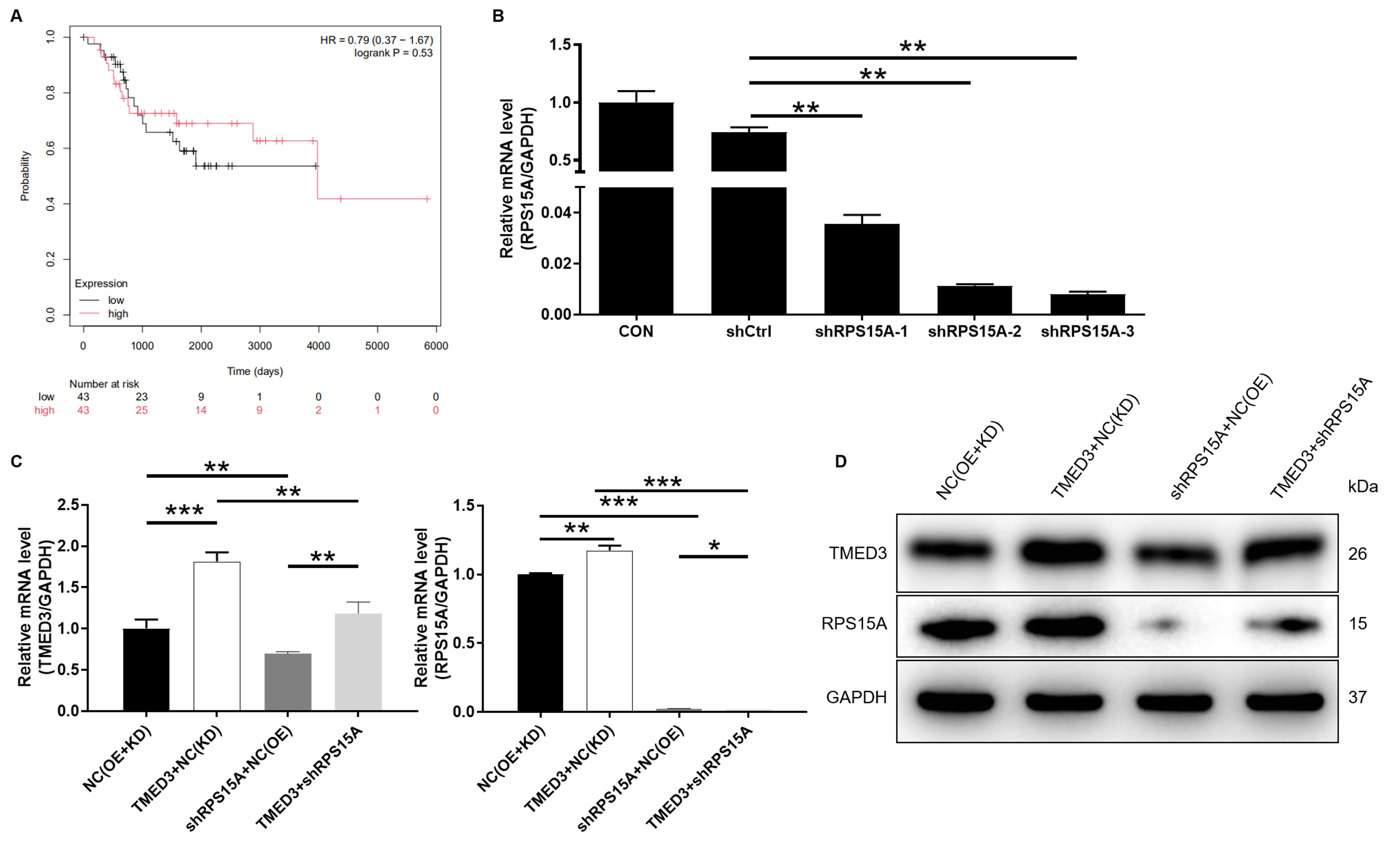

Supplement: Supplementary file 3 — Additional file 3: Figure S3. The effect of the expression level of RPS15A on the survival of osteosarcoma and the establishment of osteosarcoma cells that knock down RPS15A and TMED3 overexpression. [file 12935_2021_2340_MOESM3_ESM.tif]

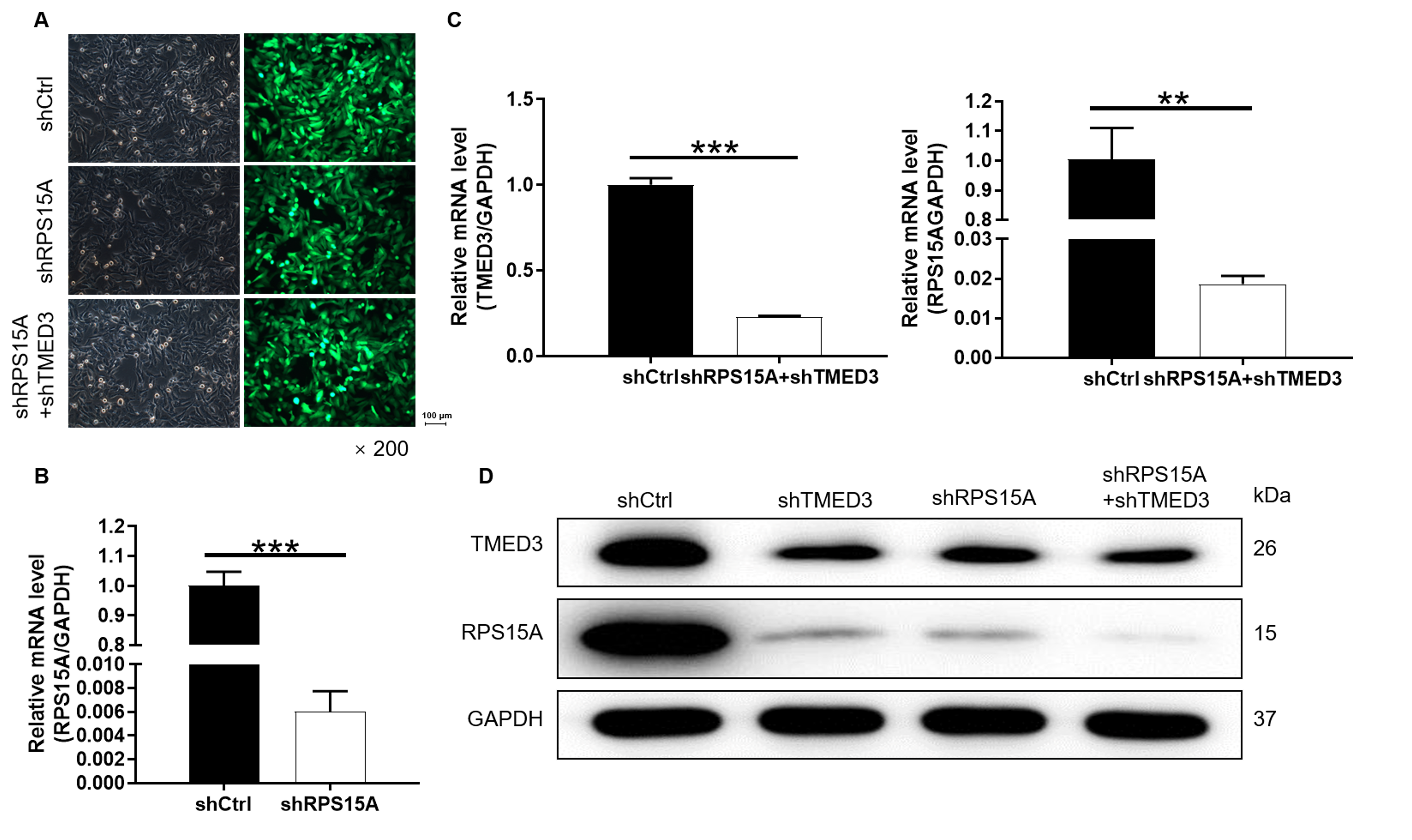

Supplement: Supplementary file 4 — Additional file 4: Figure S4. Establishment of osteosarcoma cells knocked down RPS15A and TMED3. [file 12935_2021_2340_MOESM4_ESM.tif]

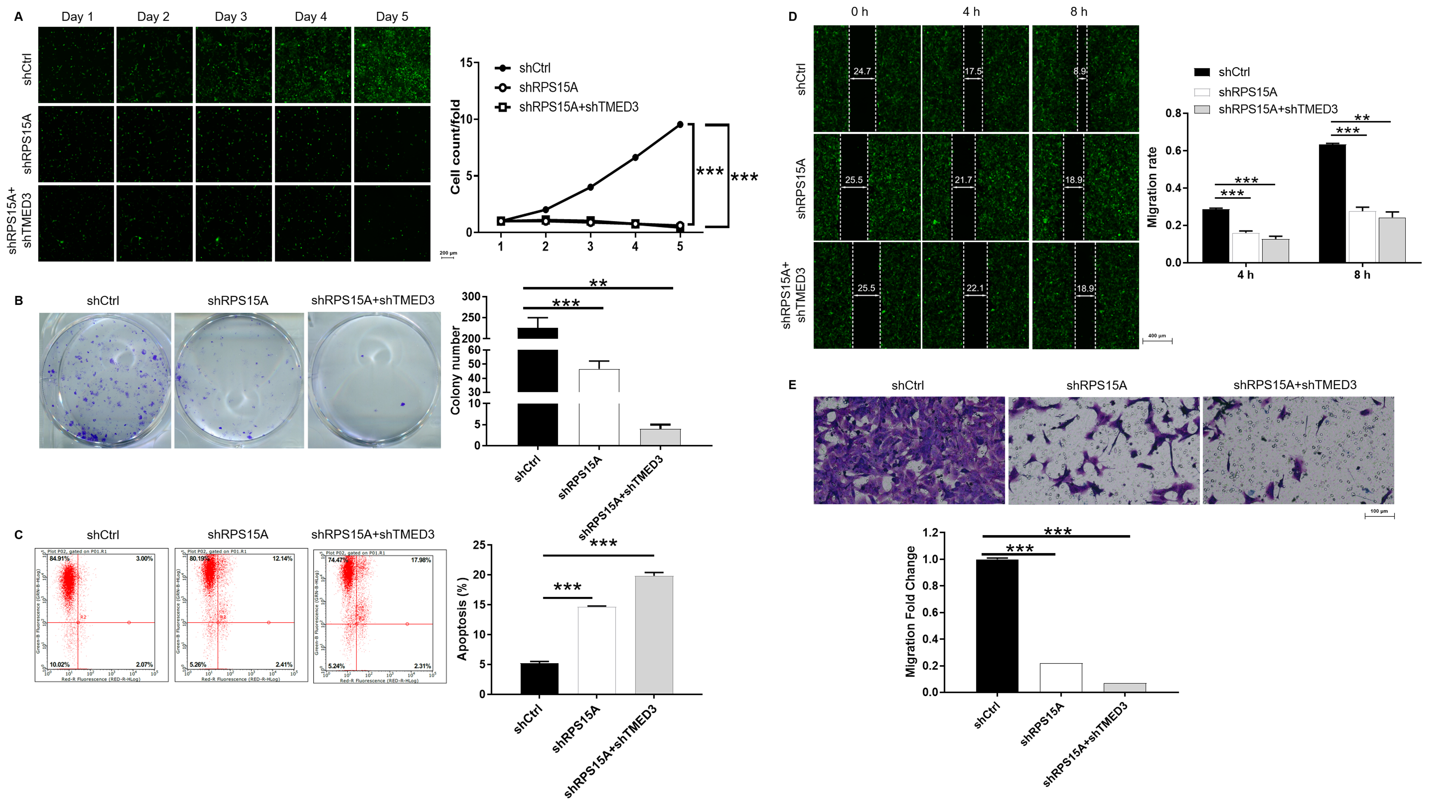

Supplement: Supplementary file 5 — Additional file 5: Figure S5. The effect of knockdown of RPS15A and TMED3 on the proliferation, apoptosis and migration of osteosarcoma cells. [file 12935_2021_2340_MOESM5_ESM.tif]
