# Supplementary material for: TMED3/RPS15A Axis promotes the development and progression of osteosarcoma
Source: Cancer Cell Int. 2021 Nov 27;21:630. doi: 10.1186/s12935-021-02340-w (PMC8626936; doi:10.1186/s12935-021-02340-w)
Supplement: Supplementary file 6 — Additional file 6. Antibodies used in IHC. Antibodies used in WB. [file 12935_2021_2340_MOESM6_ESM.docx]

Antibodies used in IHC

| Primary antibodies | Dilution in IHC | Source species | Company | Catalog No. |
| --- | --- | --- | --- | --- |
| TMED3 | 1:20 | Rabbit | abcam | ab151056 |
| RPS15A | 1:100 | Rabbit | Invitrogen | PAS-51314 |
| Ki67 | 1:400 | Rabbit | abcam | ab6721 |
| Secondary antibody | Dilution |  | Company | Catalog No. |
| HRP Goat Anti-Rabbit IgG | 1:200 |  | Abcam | Ab111909 |

Antibodies used in WB

| Antibody Name | Band Size (KDa) | Diluted Multiples | Antibody Source | Company | Number |
| --- | --- | --- | --- | --- | --- |
| TMED3 | 25 | 1: 2000 | Rabbit | Abcam | ab223175 |
| ITGA6 | 127 | 1: 2000 | Rabbit | Novus | NBP1-85747 |
| KIF20A | 100 | 1: 2000 | Rabbit | Abcam | ab70791 |
| PTGS2 | 69 | 1: 1000 | Rabbit | Abcam | ab255420 |
| RPL31 | 14 | 1: 1000 | Rabbit | Abcam | ab229534 |
| RPS15A | 15 | 1: 750 | Rabbit | Invitrogen | PA5-51314 |
| GAPDH | 37 | 1: 3000 | Rabbit | Bioworld | AP0063 |

| Secondary antibody | Dilution |  | Company | Catalog No. |
| --- | --- | --- | --- | --- |
| HRP Goat Anti-Rabbit IgG | 1:3000 |  | Beyotime | A0208 |
