# Supplementary material for: TMED3/RPS15A Axis promotes the development and progression of osteosarcoma
Source: Cancer Cell Int. 2021 Nov 27;21:630. doi: 10.1186/s12935-021-02340-w (PMC8626936; doi:10.1186/s12935-021-02340-w)
Supplement: Supplementary file 7 — Additional file 7. The target sequences and shRNA sequences. [file 12935_2021_2340_MOESM7_ESM.docx]

The target sequences and shRNA sequences

| Target | Serial Number | Target Sequence (5’-3’) |
| --- | --- | --- |
| Human-TMED3-1 | Pbr10331 | CTCTCACAAGACCGTCTACTT |
| Human-TMED3-2 | Pbr00189 | CACCTTCGAGCTGCCGGACAA |
| Human-TMED3-3 | Pbr00190 | CGTGAAGTTCTCCCTGGATTA |
| Human-RPS15A-1 | Pbr00142 | GTGCAACTCAAAGACCTGGAA |
| Human-RPS15A-2 | Pbr00143 | GCGCATGAATGTCCTGGCAGA |
| Human-RPS15A-3 | Pbr00144 | GATGACCACAGAGCTGGGAAA |
