# Supplementary material for: TMED3/RPS15A Axis promotes the development and progression of osteosarcoma
Source: Cancer Cell Int. 2021 Nov 27;21:630. doi: 10.1186/s12935-021-02340-w (PMC8626936; doi:10.1186/s12935-021-02340-w)
Supplement: Supplementary file 8 — Additional file 8. Primersused in qPCR. [file 12935_2021_2340_MOESM8_ESM.docx]

Primers used in qPCR

| Primer  Name | Upstream Primer  Sequence (5’-3’) | Downstream Primer  Sequence (5’-3’) |
| --- | --- | --- |
| GAPDH | TGACTTCAACAGCGACACCCA | CACCCTGTTGCTGTAGCCAAA |
| TMED3 | GGCGTGAAGTTCTCCCTGGATT | GCTGTCGTACTGCTTCTTCGTTTC |
| COG2 | ACCGCGTATAGCTGGCATT | CCCGTGTCTTGTCAATCGTG |
| HSPA4L | GCTTCATGGGCGATCATTT | CTTCCTCTAAGTACCGCACCTT |
| HSPA8 | CCCGAGGTGTTCCTCAGATT | CGGCCCTTGTCATTAGTGATAGTA |
| HSPA13 | TTTTACCGCAGAAGAGTTGGAG | TCTGGGGACACTGTGATGGT |
| KIF20A | GGCCGTTCCTGCATGATTGT | TGTCTGCCTTAGCCCCTTTCT |
| KIF20B | CAGCAAGATGAACCACCAGC | CGGTCGCACTTCTTCACTTTT |
| KIF23 | TTAACGCCTGGGAGGAGATAC | GCATGATGGCAAAGGTGGA |
| MAPK3 | ATTGTGCAGGACCTGATGGA | ACGTTGGCGGAGTGGATGTA |
| MAPK9 | CTCTGCGTCACCCATACATCA | TCTTTCTTCCAACTGGGCATC |
| MMP1 | ACGATTCGGGGAGAAGTGATG | TGTCGGCAAATTCGTAAGCAG |
| MYC | CATACATCCTGTCCGTCCAAG | CAAGAGTTCCGTAGCTGTTCAA |
| NEDD4 | TCCTCGGTTGGAGAATGTAGC | ACAGTTGCTCGGCGAAGTT |
| PIK3CD | GTGAACGGCAGGCATGAGTA | AGGATGGAGGAGGAATGGAC |
| PLAC8 | CACATTTTGTTTCCCGTGCCT | TCATTGCGACGCTTGTTCC |
| PRKCA | CTTCAGACAAAGACCGACGAC | CATCAGCTCCGAAACTCCAA |
| PTGS2 | CAAATCCTTGCTGTTCCCACC | TTTCTCCATAGAATCCTGTCCG |
